# C_60_ Fullerene Promotes Restoration of Kidney Function After Chronic Glyphosate Intoxication

**DOI:** 10.3390/molecules31152697

**Published:** 2026-08-03

**Authors:** Olga Abramchuk, Dmytro Nozdrenko, Svitlana Prylutska, Illia Pronko, Mykola Maliuk, Igor Vareniuk, Vsevolod Cherepanov, Olha Korzhyk, Uwe Ritter, Yuriy Prylutskyy, Vasil M. Garamus

**Affiliations:** 1 ESC “Institute of Biology and Medicine”, Taras Shevchenko National University of Kyiv, 01601 Kyiv, Ukraine; abramchuk.olga@vnu.edu.ua (O.A.); ddd@univ.kiev.ua (D.N.); psvit_1977@ukr.net (S.P.); vareniuk_igor@yahoo.com (I.V.); prylut@ukr.net (Y.P.); 2 Faculty of Biology and Forestry, Lesya Ukrainka Volyn National University, 43025 Lutsk, Ukraine; korzhyk.olha@vnu.edu.ua; 3 Kyiv Agrarian University of the NAAS of Ukraine, 01010 Kyiv, Ukraine; 4 Faculty of Veterinary Medicine, National University of Life and Environmental Science of Ukraine, 03041 Kyiv, Ukraine; illiapronkovet@gmail.com (I.P.); nikolai_malyuk@ukr.net (M.M.); 5 Institute of Physics, NAS of Ukraine, 03028 Kyiv, Ukraine; vvch2000@ukr.net; 6 Institute of Chemistry and Biotechnology, Technical University of Ilmenau, 98693 Ilmenau, Germany; uwe.ritter@tu-ilmenau.de; 7 Helmholtz-Zentrum Hereon, Institute of Metallic Biomaterials, 21502 Geesthacht, Germany

**Keywords:** C_60_ fullerene, glyphosate, renal dysfunction, biochemical analysis, histological analysis

## Abstract

Glyphosate is one of the most widely used herbicides in modern agriculture. Toxicological studies have demonstrated that chronic glyphosate exposure is associated with the development of systemic disorders, particularly renal injury. Therefore, the search for effective therapeutic approaches to mitigate glyphosate-induced kidney dysfunction remains an important challenge in contemporary biomedicine. The aim of the present study was to evaluate the effects of C_60_ fullerenes, as potent antioxidants, on the recovery of renal function following chronic glyphosate intoxication. The experiment was conducted on male Wistar rats that received glyphosate orally at a dose of 10 mg/kg body weight daily for 16 weeks. Following the cessation of glyphosate exposure, animals in the experimental group were treated with a C_60_ fullerene aqueous solution (C_60_FAS) at a dose of 1 mg/kg body weight daily for two weeks. The animals exhibited elevated blood creatinine and urea concentrations, a reduced glomerular filtration rate, increased fractional excretion of sodium, an electrolyte imbalance, and enhanced activities of superoxide dismutase and catalase after chronic glyphosate exposure. Therapeutic administration of C_60_FAS contributed to an average improvement of 20 ± 2% in the investigated biochemical parameters at the end of the experiment, which is consistent with the findings of the histological analysis of kidney tissue. These results demonstrate the pronounced therapeutic effect of C_60_ fullerenes, attributable to their ability to attenuate oxidative stress and promote the recovery of the filtration, tubular, and metabolic functions of the kidneys following chronic glyphosate intoxication.

## 1. Introduction

Glyphosate [*N*-(phosphonomethyl)glycine] is one of the most widely used non-selective herbicides in both industrial agriculture and the domestic sector [1]. Its herbicidal activity in plants is mediated through the inhibition of 5-enolpyruvylshikimate-3-phosphate synthase, a key enzyme in the shikimate pathway responsible for the biosynthesis of aromatic amino acids. Although this metabolic pathway is absent in mammals, accumulating in vivo toxicological evidence indicates that chronic glyphosate exposure, even at relatively low doses, may lead to the development of systemic disorders, including hepatic injury, neurotoxicity, endocrine dysfunction, and, most notably, renal damage [2].

The kidneys are among the primary target organs for xenobiotic-induced toxicity as they play a central role in blood filtration, metabolite excretion, and the maintenance of water and electrolyte homeostasis [3]. Chronic glyphosate intoxication has been shown to induce glomerular injury, degenerative changes in the proximal tubular epithelium, impaired mitochondrial metabolism, and activation of oxidative stress pathways [4].

It should be noted that most studies have primarily focused on the direct toxic effects of glyphosate during the exposure period, whereas the reversibility of glyphosate-induced renal alterations following the cessation of exposure remains insufficiently investigated. Analyzing the recovery process is essential for understanding the compensatory and adaptive capacity of the kidneys after chronic intoxication. Even after the toxic insult has been removed, renal dysfunction may persist for an extended period as a consequence of residual nephron damage, mitochondrial dysfunction, chronic inflammation, and sustained oxidative stress [5].

At present, the search for effective strategies to mitigate the adverse consequences of chronic glyphosate exposure remains an important challenge in biomedicine. In this context, compounds with antioxidant properties have attracted considerable interest, as excessive generation of reactive oxygen species (ROS) and the resulting oxidative stress are recognized as key pathogenic mechanisms underlying glyphosate-induced toxicity [3,6,7]. Excessive ROS accumulation disrupts intracellular redox homeostasis, damages cellular membranes, alters protein metabolism, and impairs cellular function. These events are accompanied by dysregulation of enzymatic components of the antioxidant defense system, thereby promoting the progression of tissue injury and the development of functional impairments in internal organs [8,9]. The use of antioxidant compounds has been shown to attenuate the toxic effects of glyphosate [10], reducing nephrotoxicity and hepatotoxicity, improving renal filtration function, lowering serum creatinine and urea concentrations, and alleviating structural alterations in affected tissues [3].

Among antioxidant agents, carbon nanomaterials, particularly C_60_ fullerenes, have attracted considerable attention because of their remarkable capacity to scavenge ROS [11]. In vivo studies have demonstrated that C_60_ fullerenes reduce free radical oxidation and preserve the activity of endogenous antioxidant defense systems [12,13]. Their high biocompatibility, relatively low toxicity, and ability to penetrate biological barriers [12,14,15,16] make water-soluble C_60_ fullerenes a promising therapeutic approach for the management of pathological conditions associated with oxidative stress.

Accordingly, the aim of the present study was to evaluate the effects of C_60_ fullerenes, as potent antioxidants, on nephrotoxicity development in rats following prolonged glyphosate exposure by assessing selected biochemical markers of renal injury and the histomorphological characteristics of renal tissue.

## 2. Results and Discussions

### 2.1. Atomic Force Microscopy (AFM) Research

**Figure 1:**
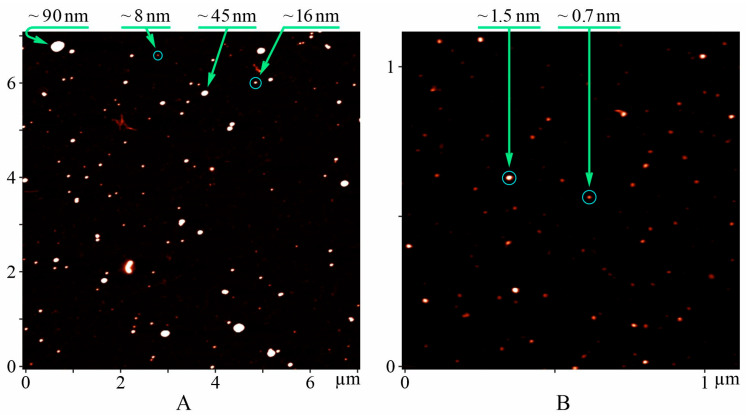
AFM images (**A**,**B**) of a С_60_ fullerene layer deposited from C_60_FAS on a mica substrate. Arrows indicate the height of the nano-objects.

The structure of the C_60_ fullerene layer consists of dot-like and circular features ranging in height from a few nm to 100 nm (Figure 1A). They correspond to spherical aggregates characteristic of C_60_ fullerene aqueous solutions (C_60_FASs) in a highly aggregated form. The size distribution lacks a well-defined maximum; however, the diameters of most particles fall within the range of 10–50 nm. To visualize particles smaller than 5 nm, high-magnification AFM images were recorded over sample areas free of large aggregates. These images (Figure 1B) show dot-like features with a height of ~0.7 nm, which correspond to individual C_60_ molecule. Furthermore, a small number of aggregates with a diameter of ~1.5 nm are present. Thus, the AFM data reveal that C_60_ fullerenes in the water solution exist in both molecular and highly aggregated polydisperse forms, which agrees well with our early research [17]. The detected nanoformation of the C_60_ molecules in C_60_FAS indicates their suitability for in vivo testing.

### 2.2. Biochemical Research

A number of experimental studies [18,19,20,21] have demonstrated that chronic glyphosate administration is associated with elevated serum creatinine and urea concentrations, a reduced glomerular filtration rate (GFR), impaired electrolyte transport, and altered activities of antioxidant enzymes, indicating the development of glyphosate-induced nephrotoxicity.

Elevated blood creatinine concentration is one of the most informative biomarkers of renal dysfunction associated with chronic glyphosate exposure [22]. An increase in this parameter reflects impaired glomerular filtration and indicates a decline in the excretory capacity of the nephrons [23]. Excessive generation of ROS destabilizes cellular membranes, disrupts the integrity of the tubular epithelium, and impairs tubular reabsorptive function.

**Figure 2:**
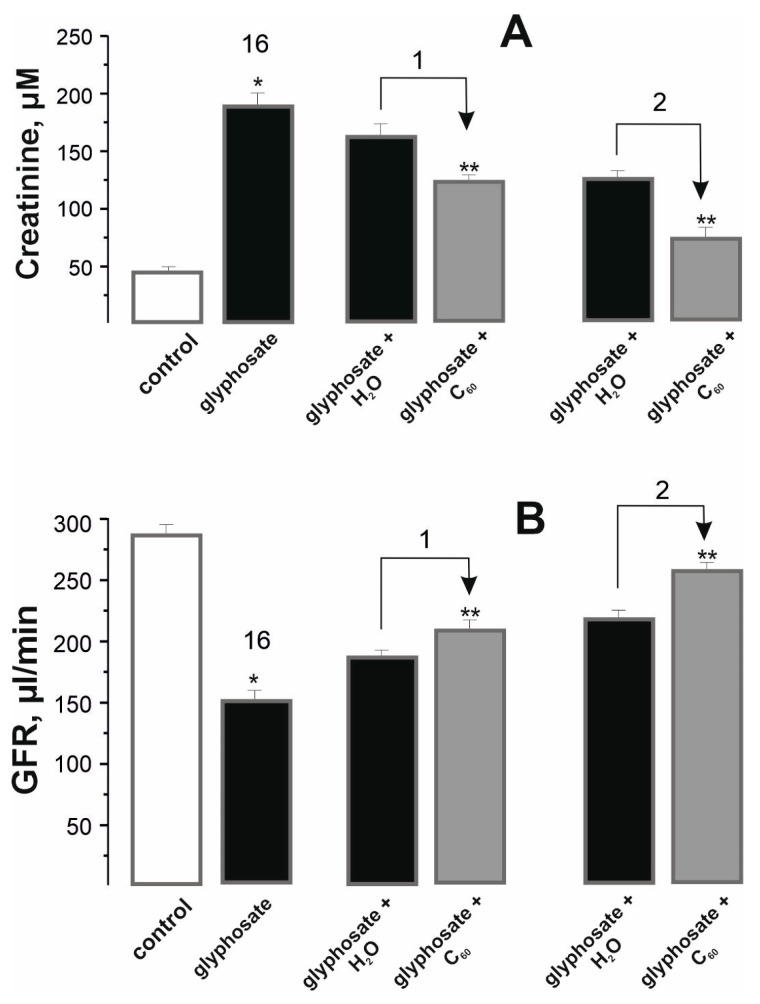
Blood creatinine concentration (**A**) and glomerular filtration rate (GFR) (**B**) in rats: control (n = 10), after glyphosate intoxication (16 weeks glyphosate; n = 10), after cessation of glyphosate intoxication and treatment with distilled water (1 week glyphosate + H_2_O (n = 10) and 2 weeks glyphosate + H_2_O (n = 10)), and after cessation of glyphosate intoxication and treatment with C_60_FAS (1 week glyphosate + C_60_ (n = 10) and 2 weeks glyphosate + C_60_ (n = 10)). * *p* < 0.05 versus the control group; ** *p* < 0.05 versus the glyphosate + H_2_O group.

The obtained results (Figure 2A) demonstrated a significant increase in blood creatinine concentration following 16 weeks of glyphosate intoxication, reaching 182 ± 4 μM compared with the control value of 49 ± 1 μM. Treatment with C_60_FAS during the first and second weeks after the cessation of glyphosate exposure resulted in a marked reduction in creatinine concentration compared with animals receiving distilled water. During the first week, this parameter decreased to 124 ± 3 μM in the C_60_FAS-treated group, compared with 161 ± 4 μM in the untreated group. By the end of the second week, the creatinine concentration further declined to 73 ± 2 μM in C_60_FAS-treated animals, whereas it remained at 125 ± 3 μM in animals receiving distilled water. Accordingly, the therapeutic efficacy of C_60_FAS reached 23 ± 1% and 42 ± 2% at the end of the first and second weeks of treatment, respectively.

The GFR value is widely used in both experimental and clinical nephrology as the primary indicator of renal filtration function and the degree of nephron impairment [24]. A reduction in GFR during chronic glyphosate intoxication has been attributed to glomerular injury, oxidative stress, impaired renal microcirculation, and inflammatory changes in renal tissue [25]. An additional contributor to the decline in glomerular filtration may be rhabdomyolysis-associated kidney injury, which is characterized by the formation of myoglobin casts and partial obstruction of the tubular lumen [26].

As shown in Figure 2B, GFR was significantly reduced after 16 weeks of glyphosate administration, reaching 150 ± 3 μL/min compared with 282 ± 3 μL/min in the control group. Treatment with C_60_FAS accelerated the recovery of GFR following the cessation of glyphosate exposure. By the end of the first week of treatment, GFR increased to 213 ± 3 μL/min, compared with 181 ± 4 μL/min in animals receiving distilled water. At the end of the second week, GFR further increased to 256 ± 3 μL/min in the C_60_FAS-treated group, whereas it reached only 212 ± 5 μL/min in the corresponding untreated group. Accordingly, the nephroprotective efficacy of C_60_FAS was estimated to be 15 ± 1% and 17 ± 1% at the end of the first and second weeks of treatment, respectively.

The improvement in the above-mentioned parameters following C_60_FAS treatment may be attributed to the ability of C_60_ fullerenes to scavenge ROS, thereby promoting the restoration of nephron structural integrity.

Chronic glyphosate intoxication is characterized by impaired renal excretory function, accompanied by the accumulation of nitrogenous waste products in the bloodstream [4]. These alterations result from damage to the structural components of the nephron, particularly the glomerular apparatus and the tubular epithelium, leading to a reduction in GFR and impaired elimination of metabolic waste products. Among the biochemical indicators of renal dysfunction, blood urea concentration is considered one of the most informative biomarkers of these pathological changes [27]. Toxic injury to renal tissue induced by glyphosate compromises glomerular filtration, thereby reducing urea excretion and resulting in its progressive accumulation in the blood [23].

**Figure 3:**
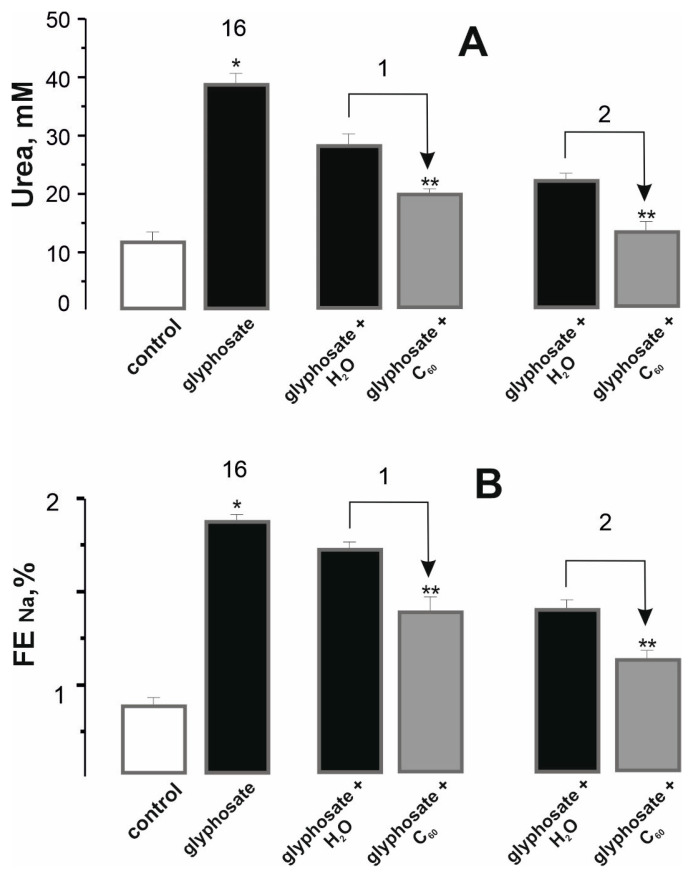
Blood urea concentration (**A**) and fractional excretion of sodium (FE_Na_) (**B**) in rats: control (n = 10), after glyphosate intoxication (16 weeks glyphosate (n = 10)), after cessation of glyphosate intoxication and treatment with distilled water (1 week glyphosate + H_2_O (n = 10) and 2 weeks glyphosate + H_2_O (n = 10)), and after cessation of glyphosate intoxication and treatment with C_60_FAS (1 week glyphosate + C_60_ (n = 10) and 2 weeks glyphosate + C_60_ (n = 10)). * *p* < 0.05 versus the control group; ** *p* < 0.05 versus the glyphosate + H_2_O group.

As shown in Figure 3A, the blood urea concentration increased to 37 ± 2 mM after 16 weeks of glyphosate intoxication, compared with the control value of 12 ± 1 mM. Treatment with C_60_FAS reduced the urea concentration to 20 ± 1 and 14 ± 1 mM at the end of the first and second weeks following the cessation of glyphosate exposure, respectively. In contrast, urea concentrations remained at 28 ± 1 and 22 ± 1 mM in the corresponding groups receiving distilled water. Accordingly, the therapeutic efficacy of C_60_FAS was 29 ± 1% and 36 ± 2% at the end of the first and second weeks of treatment, respectively.

In glyphosate-induced nephrotoxicity, oxidative stress and injury to the renal tubular epithelium lead to reduced Na^+^/K^+^-ATPase activity, thereby impairing tubular sodium reabsorption [28]. Consequently, urinary sodium excretion increases, as reflected by an elevated fractional excretion of sodium (FE_Na_), a sensitive biomarker of tubular dysfunction [29].

As shown in Figure 3B, the FE_Na_ increased significantly from 0.8 ± 0.1% in the control group to 1.9 ± 0.1% after 16 weeks of glyphosate intoxication. Treatment with C_60_FAS reduced FE_Na_ to 1.4 ± 0.1% and 1.2 ± 0.1% at the end of the first and second weeks following the cessation of glyphosate exposure, respectively. In contrast, the FE_Na_ value remained at 1.7 ± 0.1% and 1.4 ± 0.1% in the corresponding groups treated with distilled water. Accordingly, the nephroprotective efficacy of C_60_FAS was 18 ± 1% and 14 ± 1% at the end of the first and second weeks of treatment, respectively.

The kidneys play a central role in maintaining body fluid and electrolyte homeostasis. They regulate the concentrations of sodium (Na^+^) and potassium (K^+^) ions in the blood through glomerular filtration, tubular reabsorption, and tubular secretion. Sodium is essential for maintaining the extracellular fluid volume and osmotic balance [30], whereas potassium is required for the generation of membrane potential, nerve impulse conduction, muscle contraction, and normal cardiovascular function [31]. Therefore, disturbances in the concentrations of these electrolytes represent important indicators of renal dysfunction and reflect impairments in both glomerular filtration and tubular function.

**Figure 4:**
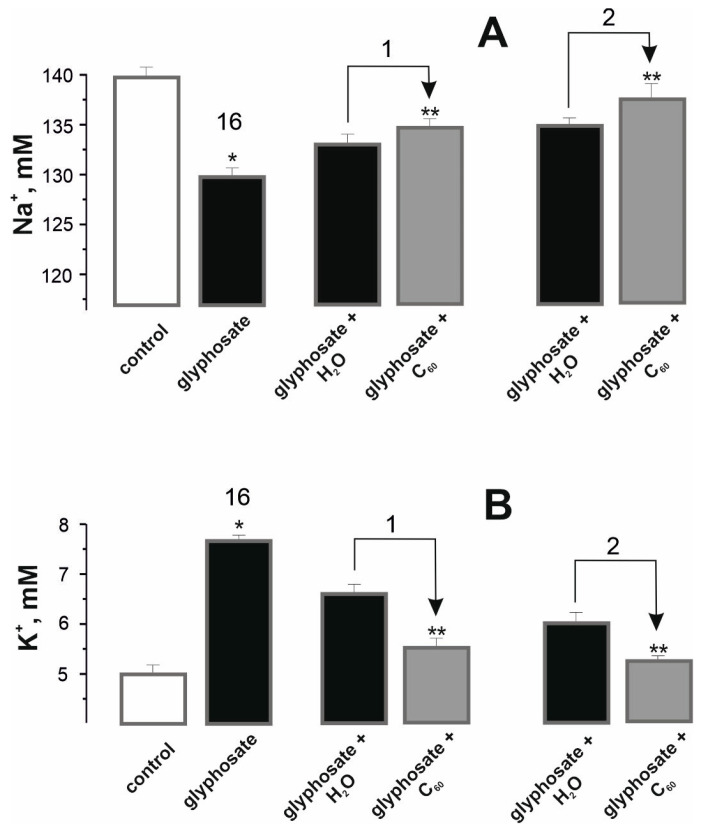
Blood sodium (Na^+^) (**A**) and potassium (K^+^) (**B**) concentrations in rats: control (n = 10), after glyphosate intoxication (16 weeks glyphosate (n=10)), after cessation of glyphosate intoxication and treatment with distilled water (1 week glyphosate + H_2_O (n = 10) and 2 weeks glyphosate + H_2_O (n = 10)), and after cessation of glyphosate intoxication and treatment with C_60_FAS (1 week glyphosate + C_60_ (n = 10) and 2 weeks glyphosate + C_60_ (n = 10)). *p* < 0.05, * *p* < 0.05 versus the control group; ** *p* < 0.05 versus the glyphosate + H_2_O group.

As shown in Figure 4A, the Na^+^ concentration decreased to 129.3 ± 3 mM after 16 weeks of glyphosate intoxication, compared with the control value of 140.7 ± 2 mM. Thus, the Na^+^ concentration decreased by 8±1% relative to the control throughout the experimental period. It is important to note that a 10% reduction in blood sodium concentration corresponds to clinically significant hyponatremia, which is associated with substantial nephron dysfunction and an increased risk of systemic and neurological complications [32].

Treatment with C_60_FAS increased this parameter to 134.7 ± 3 and 138.2 ± 4 mM at the end of the first and second weeks, respectively, compared with 131.3 ± 3 and 134.6 ± 3 mM in the corresponding groups without C_60_FAS treatment. Thus, the therapeutic effect of C_60_FAS was approximately 3 ± 1% in both cases, which is a significant value [32].

As shown in Figure 4B, the K^+^ concentration increased to 7.7 ± 0.3 mM after 16 weeks of glyphosate intoxication, compared with the control value of 5.1 ± 0.4 mM. Thus, the K^+^ concentration increased by 34 ± 1% relative to the control throughout the experimental period. It is important to note that a 20% increase in blood potassium concentration in the setting of renal insufficiency is considered a severe pathological condition as potassium accumulation can rapidly disrupt the electrical activity of the heart [33].

After the first and second weeks of C_60_FAS treatment, this parameter was 5.5 ± 0.3 and 5.3 ± 0.3 mM, respectively, compared with 6.6 ± 0.3 and 6.1 ± 0.3 mM in the corresponding groups without C_60_FAS treatment. Thus, the therapeutic effect of C_60_FAS was 17 ± 1% and 13 ± 1% at the end of the first and second weeks of the experiment, respectively.

The decrease in blood sodium concentration and the increase in blood potassium concentration during chronic glyphosate intoxication may be associated with impaired reabsorptive and secretory functions of the renal tubules, suppression of Na^+^/K^+^-ATPase activity, reduced GFR, and the development of nephrotoxic changes in the renal tissue [34]. By reducing the intensity of oxidative stress and limiting damage to cellular membranes, water-soluble C_60_ fullerenes promote the preservation of Na^+^/K^+^-ATPase functional activity, reduce injury to the renal tubular epithelium, and improve electrolyte reabsorption. As a result, a trend toward normalization of blood sodium and potassium concentrations was observed, indicating attenuation of glyphosate-induced nephrotoxicity and partial restoration of renal function.

The enzymes of the antioxidant defense system, primarily superoxide dismutase (SOD) and catalase (CAT), play an important role in maintaining the prooxidant–antioxidant balance. SOD mediates the first stage of antioxidant defense by neutralizing superoxide anion radicals and converting them into hydrogen peroxide, which is subsequently decomposed by CAT into water and oxygen. During acute glyphosate intoxication, SOD activity is generally reduced as a result of pronounced oxidative damage [5]. In contrast, chronic exposure to moderate doses of glyphosate is associated with increased SOD activity, which is considered a compensatory response aimed at limiting excessive ROS production and maintaining the antioxidant defense system [35].

**Figure 5:**
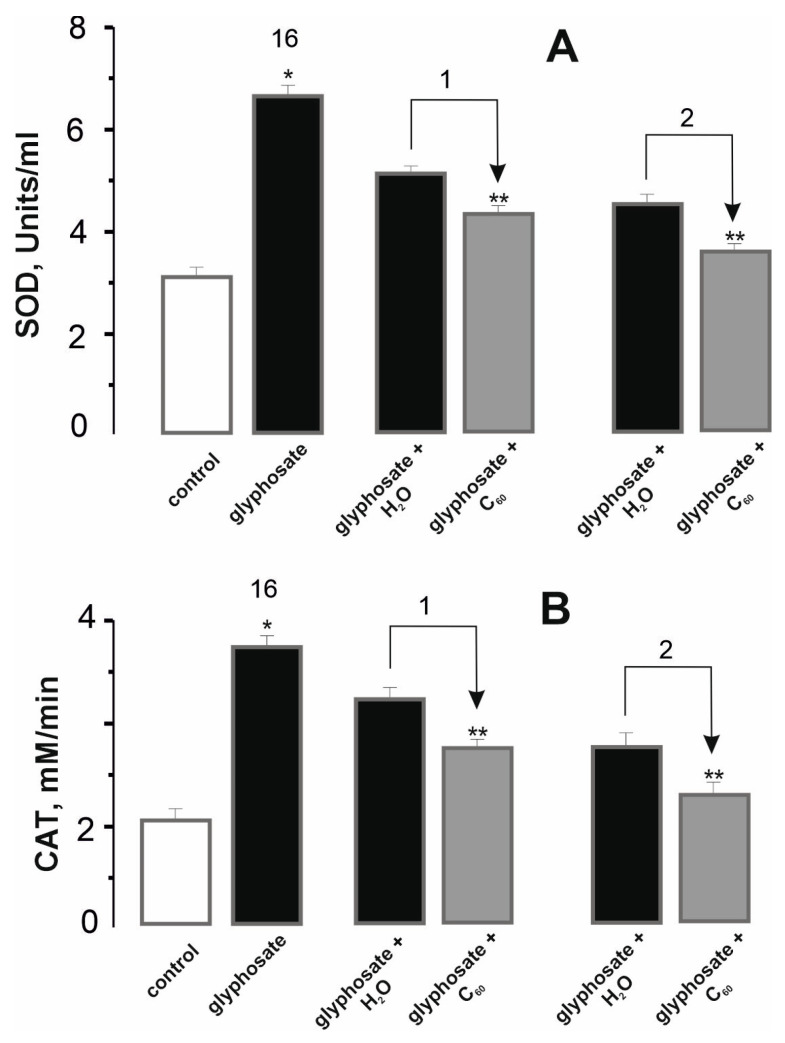
Activities of SOD (**A**) and CAT (**B**) in rat blood: control (n = 10), after glyphosate intoxication (16 weeks glyphosate (n = 10)), after cessation of glyphosate intoxication and treatment with distilled water (1 week glyphosate + H_2_O (n = 10) and 2 weeks glyphosate + H_2_O (n = 10)), and after cessation of glyphosate intoxication and treatment with C_60_FAS (1 week glyphosate + C_60_ (n = 10) and 2 weeks glyphosate + C_60_ (n = 10)). * *p* < 0.05 versus the control group; ** *p* < 0.05 versus the glyphosate + H_2_O group.

In the experiment, an increase in SOD activity was recorded after the 16th week of chronic glyphosate administration, from 2.5 ± 0.1 Units/mL (control) to 6.7 ± 0.3 Units/mL (Figure 5A). During the first week of C_60_FAS treatment, this parameter was 4.3 ± 0.2 Units/mL, and during the second week it was 3.8 ± 0.2 Units/mL, compared with 5.1 ± 0.2 and 4.4 ± 0.2 Units/mL, respectively, in the groups without C_60_FAS treatment. Thus, the therapeutic effect of C_60_FAS was 16 ± 1% and 14 ± 1% at the end of the first and second weeks of the experiment, respectively.

During prolonged glyphosate exposure, excessive ROS production initiates the activation of intracellular antioxidant defense mechanisms, including the Nrf2 signaling pathway [13]. Under these conditions, the increase in SOD activity can be regarded as an adaptive response aimed at neutralizing excessive free radicals and reducing the severity of oxidative stress. As shown by the obtained data, the use of the antioxidant C_60_ fullerene promotes the attenuation of free radical processes and supports the activity of the endogenous antioxidant defense system.

CAT is an important enzyme of the antioxidant defense system that prevents the accumulation of hydrogen peroxide in cells. The enhancement of oxidative stress during chronic glyphosate intoxication is accompanied by an increase in CAT activity, reflecting the compensatory activation of the antioxidant defense system aimed at limiting free radical-induced tissue damage [36].

In the experiment, an increase in CAT activity was recorded after the 16th week of glyphosate intoxication to 3.8 ± 0.3 mM/min, compared with the control value of 2.1 ± 0.2 mM/min (Figure 5B). Treatment with C_60_FAS during the first and second weeks reduced this parameter to 2.7 ± 0.2 and 2.3 ± 0.2 mM/min, respectively, compared with 3.3 ± 0.2 and 2.7 ± 0.2 mM/min in the corresponding groups without C_60_FAS treatment. Thus, the therapeutic effect of C_60_FAS was 18 ± 1% and 15 ± 1% at the end of the first and second weeks of the experiment, respectively.

Thus, therapeutic administration of C_60_FAS was accompanied by an improvement in the studied blood biochemical parameters in rats by an average of 20 ± 2% at the end of the second week of the experiment. It can be assumed that this effect is attributable to the antioxidant properties of C_60_ fullerene, which contribute to reducing the severity of oxidative stress and maintaining the activity of the endogenous antioxidant defense system. These results are consistent with our recent study [37], which showed that water-soluble C_60_ fullerenes attenuate signs of acute renal failure in rats undergoing rhabdomyolysis by inhibiting oxidative stress.

### 2.3. Histological Analysis of Kidney Tissue

**Figure 6:**
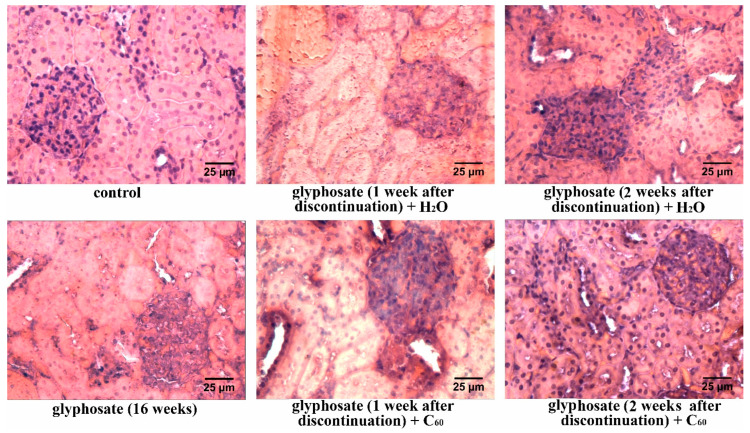
Representative histological images of the kidney in rats: control (n = 10), after glyphosate intoxication (16 weeks glyphosate (n = 10)), after cessation of glyphosate intoxication and treatment with distilled water (1 week glyphosate + H_2_O (n = 10) and 2 weeks glyphosate + H_2_O (n = 10)), and after cessation of glyphosate intoxication and treatment with C_60_FAS (1 week glyphosate + C_60_ (n = 10) and 2 weeks glyphosate + C_60_ (n = 10)). Hematoxylin and eosin staining. Scale bars—25 μm.

No deviations from the normal histological structure of the kidneys were observed in rats of the control group. After 16 weeks of the glyphosate administration, glomerular shrinkage and fibrosis, tubular necrosis, inflammation and hemorrhage in all parts of the kidney were observed (Table 1; Figure 6).

These histopathological features persisted in 1 and 2 weeks after discontinuation of the glyphosate, with only a slight decrease in intensity (Table 1; Figure 6).

The use of C_60_FAS reduced the degree of some pathohistological features: from strong to moderate, or from moderate to weak (Table 1; Figure 6). This indicates that water-soluble C_60_ fullerenes reduce the extent of glyphosate-induced kidney tissue damage, which is consistent with biochemical analysis data.

## 3. Materials and Methods

### 3.1. Preparation of C_60_FAS

C_60_FAS was prepared according the method in [38]. Briefly, we used a saturated solution of C_60_ fullerene (Sigma-Aldrich, Germany; purity > 99.95%) in toluene with a C_60_ molecule concentration corresponding to the maximum solubility near 2.9 mg/mL, and the same amount of distilled water in an open beaker. The two phases formed were treated in ultrasonic bath (8 Hz, 8 h). The procedure was continued until the toluene had completely evaporated and the water phase became yellow in color. Toluene could not be detected in the water by gas chromatography–mass spectrometry (PerkinElmer, Waltham, MA, USA) analysis [38]. The resulting C_60_FAS (maximum concentration 0.15 mg/mL) remains highly stable for 12–18 months at a temperature +4–25 °C: its high zeta potential value, −25.3 mV, indicates a low tendency for C_60_ fullerenes to aggregate in aqueous solution over time [39].

The morphological state of the C_60_ fullerene particles in C_60_FAS was studied by AFM. Sample preparation involved deposition of a microdrop of C_60_FAS onto a freshly cleaved surface of a mica substrate, followed by drying under ambient conditions. Measurements were carried out using the “Solver Pro M” system (NT-MDT, Eindhoven, The Netherlands) in the semicontact (tapping) mode with probes “RTESPA-150” (Bruker, Billerica, MA, USA). The tapping mode was used with the free cantilever oscillation amplitude set to 80 nm.

### 3.2. In Vivo Experiment

The experiments were carried out on 60 male Wistar rats that were approximately one month old and weighed 150–180 ± 5 g. The number of animals was determined based on a similar experimental study [38] and the Reduction principle in the 3Rs concept. The rats were housed under controlled environmental conditions (21 °C, 12 h light/12 h dark cycle) with ad libitum access to water and a standard diet. All procedures with laboratory animals complied with the ARRIVE guidelines. The in vivo study protocol was approved by the Biomedical Ethics Committee of the ESC “Institute of Biology and Medicine” at Taras Shevchenko National University of Kyiv (Protocol No. 4, 30 July 2024) and carried out in accordance with Article 26 of the Law of Ukraine “On the Protection of Animals from Cruelty” (No. 3447-IV, 21 February 2006), as well as the European Union Directive of 22 September 2010 (2010/63/EU) on the protection of animals used for scientific purposes.

Animals were randomly assigned to six experimental groups: in group 1 (n = 10), chronic glyphosate intoxication was induced by daily oral administration of glyphosate at a dose of 10 mg/kg body weight for 16 weeks. Following the completion of glyphosate exposure, animals in group 2 (n = 10) and group 3 (n = 10) received C_60_FAS at a dose of 1 mg/kg body weight once daily for one and two weeks, respectively. Animals in group 4 (n = 10) and group 5 (n = 10) received an equivalent volume of distilled water once daily for one and two weeks, respectively, after the cessation of glyphosate administration. Animals in the control group (group 6 (n = 10)) were maintained under identical experimental conditions and received an equivalent volume of distilled water throughout the study.

The animals’ overall health was assessed daily based on their appearance, motor activity, feeding and drinking behavior, and coat condition. During the experiments, the blood pressure and heart rate of the rats were continuously monitored using a PM5000V veterinary patient monitor (AriVetcare, Hefei City, Anhui, China).

The absence of an experimental group receiving only C_60_FAS was due to the results of our previous study [37], which showed that oral administration of C_60_FAS at a dose of 1 mg/kg does not cause statistically significant changes in biochemical parameters of renal function and is not accompanied by histopathological changes in renal tissue in healthy rats.

To model chronic intoxication, glyphosate potassium salt (Glyphosate Potassium Salt, CAS 70901-12-1, purity ≥99%; Chemicea Pharmaceuticals Pvt. Ltd., Navi Mumbai, India; Cat. No. CP-G23019) was used. The reagent was research grade and was accompanied by a Certificate of Analysis (CoA) from the manufacturer. The working solution was prepared by dissolving the required amount of the reagent in sterile distilled water immediately before administration. Glyphosate was administered to animals orally using a metal gastric tube to ensure accurate dosing.

The chosen dose of glyphosate exceeds the permissible daily dose (1 mg/kg) established by regulatory authorities, but is significantly lower than the dose that causes acute toxicity, which allowed us to study the long-term effects of chronic exposure to glyphosate [40].

It should be noted that this chronic glyphosate intoxication model in rats does not fully reproduce all the characteristics of the herbicide’s effects on the human body. Therefore, extrapolating the obtained data to clinical settings requires caution.

The dose of C_60_FAS used was selected based on previous findings demonstrating its pronounced antioxidant effects in a study on the dynamics of *musculus soleus* contraction in rats with chronic glyphosate intoxication [19]. Moreover, the cumulative dose of 14 mg/kg administered throughout the experimental period was substantially lower than the reported LD_50_ value of 721 mg/kg for intraperitoneal administration in mice [39] and was therefore considered safe for in vivo testing. Finally, recent research, conducted by a laboratory accredited by both the FDA and OECD, further confirmed that C_60_ fullerenes are not genotoxic [41].

The two-week post-exposure observation period in rats represents a physiologically meaningful interval for recovery and adaptation, which corresponds to a several-month-long post-toxic recovery period in humans. This duration is considered sufficient for the stabilization of most acute compensatory responses and enables the assessment of whether alterations in renal function are transient or progress to persistent dysfunction [42].

### 3.3. Biochemical Analysis

Creatinine, urea, sodium and potassium concentrations, as well as the activities of SOD and CAT, were used as markers of renal functional status and the prooxidant–antioxidant balance, respectively, and were determined using RNL-200 and JN-1101-TR2 clinical biochemical analyzers (Netherlands) using commercial diagnostic reagent kits (Cormay Diagnostics Sp. z o.o., Poland) in accordance with the manufacturer’s instructions.

The GFR value was determined based on the clearance of endogenous creatinine [43]:

GFR (μL/min) = (V × Cr_U_)/Cr_B_,

where V is the urine volume (μL/min; minute diuresis), and Cr_U_ (μM) and Cr_B_ (μM) are the concentrations of creatinine in urine and blood, respectively.

Note that endogenous creatinine clearance was normalized to animal body weight (per 100 g).

To assess the functional status of the renal tubular apparatus and the efficiency of sodium reabsorption processes, the FE_Na_ value was determined [44]:

FE_Na_ (%) = 100% × (Cr_B_ × Na_U_)/(Na_B_ × Cr_U_),

where Na_U_ and Na_B_ are the concentrations of sodium in urine and blood, respectively.

It should be noted that the animals were kept in laboratory cages equipped with a special urine collection tray. Urine was collected once daily, one hour after the water load.

The levels of biochemical markers of renal injury and the prooxidant–antioxidant balance were assessed at the end of each experimental week.

### 3.4. Histological Analysis

The samples of a kidney were separated and fixed in 10% formalin, embedded in paraffin, cut into 5 µm thick sections and stained with hematoxylin and eosin [45]. Six sections were made from each sample. Eight to twelve digital microphotographs of stained sections from each sample were taken at ×400 magnification using a computer-assisted image analyzing system (consisting of an Olympus BX41 microscope and Olympus C-5050 Zoom digital camera (Olympus, Tokyo, Japan)). The histopathological features of the glomerular, tubular and interstitial damage to the kidney were determined by light microscopy observation using an EGTI system with modifications. The intensity of each histopathological feature was evaluated as follows: “–”—not observed; “+”—weak; “++”—moderate; “+++”—strong. The histopathological score was calculated as the sum of all “+”.

At the end of the 16th week of the experiment, as well as at the end of the first and second experimental weeks after cessation of glyphosate intoxication and treatment with distilled water and C_60_FAS, animals from groups 1–6 were sacrificed to collect tissues for histological studies. Euthanasia of animals was conducted by administering an overdose of an anesthetic agent (Zoletil (France), 40 mg/kg).

### 3.5. Statistical Analysis

Data from six independent experimental groups (n = 10 rats per group) were analyzed for each biochemical parameter. Each rat was measured once at the end of the experiment. Prior to hypothesis testing, the data distribution was evaluated using the Shapiro–Wilk test, and homogeneity of variances across groups was assessed using Levene’s test. All parameters met the assumptions required for parametric analysis. Between-group comparisons were performed using one-way ANOVA. When a significant main effect was detected, pairwise differences between groups were examined using Tukey’s HSD post hoc test. The results are presented as the mean ± SD. Statistical significance was set at *p* < 0.05 for all analyses. All statistical procedures were carried out using Statistica 8.0 (Dell, USA).

## 4. Conclusions

The obtained results indicate that chronic glyphosate exposure leads to the development of a pronounced nephrotoxic syndrome accompanied by impairment of both the glomerular filtration and tubular functions of the kidneys. The main manifestations of this toxic injury include increased blood creatinine and urea concentrations, reduced GFR, increased FE_Na_, altered blood sodium and potassium concentrations, and changes in the activities of the key antioxidant enzymes SOD and CAT.

It was found that therapeutic administration of C_60_FAS following the cessation of chronic glyphosate intoxication was accompanied by an improvement in the investigated biochemical parameters, averaging 20 ± 2% by the end of the treatment period, which was confirmed by the findings of the histological analysis of renal tissue.

These findings suggest that the therapeutic effect of water-soluble C_60_ fullerenes is primarily attributable to their pronounced antioxidant properties, which are attributed to the ability of these carbon nanomaterials to preserve the structural and functional integrity of nephrons. This indicates the potential of C_60_ fullerenes in correcting the toxic kidney injury associated with enhanced free radical processes, but requires further preclinical testing.

## Figures and Tables

**Table 1:** Histopathological signs of kidney damage in rats after treatment with glyphosate and C_60_FAS.

	Histopathological Sign	Glomerular			Tubular			Interstitial		
Group		Glomerular Inflammation and Hemorrhage	Retraction of Glomerular Tuft	Glomerular Fibrosis	Loss of Brush Border and Thickening of Basement Membrane	Tubular Inflammation and Cast Formation	Tubular Necrosis	Inflammation	Hemorrhage	Interstitial Necrosis
Control		–	–	–	–	–	–	–	–	–
Glyphosate (16 weeks)		+++	++	++	+++	+++	++	+++	+++	+++
Glyphosate (1 week after discontinuation) + H_2_O		+++	++	+++	+++	+++	++	+++	+++	+++
Glyphosate (1 week after discontinuation) + C_60_		++	+	++	+++	++	+	+++	++	++
Glyphosate (2 weeks after discontinuation) + H_2_O		+++	++	+	+++	++	+	++	++	++
Glyphosate (2 weeks after discontinuation) + C_60_		++	++	+	++	+	+	++	++	+

Trait intensity: “–”—not observed; “+”—weak; “++”—moderate; “+++”—strong.

## Data Availability

Dataset available upon request from the authors.
